# Report of the Sanitary Commission of Massachusetts, 1850

**Published:** 1851-05

**Authors:** 


REPORT OF THE SANITARY COMMISSION OF MASSACHUSETTS, 1850.

Through the polite attention of our friends Dr. Edward Jarvis, of Dorchester, and Lemuel Shattuck, Esq., of Boston, Mass., we have received a copy of a *Report of a general plan for the promotion of public and personal health, devised, prepared and recommended by the Commissioners appointed under a resolve of the Legislature of Massachusetts, relating to a sanitary survey of the State.* Our readers are aware of the deep interest we feel in everything relating to the subject of this report, and they know how constantly and earnestly we have endeavored to impress upon the public mind, the vast importance of everything promotive of public and personal health. It is the one great theme that should absorb the public mind, and, if we could see the vast regions of the West and South awakened to the truths of sanitary science, and engaged in putting them into action, we should feel secure of their rapid attainment of prosperity, and permanent possession of it. But while daily experience shows the absolute necessity of a knowledge of sanitary science, years roll on without its attainment, and ignorance and time are building difficulties for the future. The profession which should be foremost in bringing about a reform, plays the sluggard, and the great public sleeps securely until pestilence has it in its jaws, and then we see spasmodic twitchings after sanitary help.

In the midst of this general apathy it is refreshing and consoling to look upon the philanthropic and noble labors of Massachusetts in sanitary reform, and it was fortunate for her, and for the cause of humanity, that she possessed such a man as Lemuel Shattuck, to be placed at the head of a commission for a sanitary survey of the State. No one in Europe or America was gifted with higher qualifications for the work, and this Report is a monument to his philanthropy and intellectual powers, more honorable than the proudest trophies of the warrior. The labors of the Massachusetts Sanitary Commission are a model, which all the States of this Union would do well in copying as speedily as possible.

We shall endeavor to present the readers of this *Journal*, in future numbers, an analysis of the contents of this whole volume, and we hope that the startling truths of this Report will awaken our professional brethren to the necessity of a study of sanitary science. The proudest names in the medicine and surgery of France have devoted themselves to gratuitous sanitary labors for years, and a host of British physicians have imitated the example, and why should American physicians hold back from this great enterprise of philanthropy? We shall certainly do all in our power to awaken the sleeping energies of the profession.

